# Regulation of a rat VL30 element in human breast cancer cells in hypoxia and anoxia: role of HIF-1

**DOI:** 10.1038/sj.bjc.6600576

**Published:** 2002-11-04

**Authors:** K Ameri, B Burke, C E Lewis, A L Harris

**Affiliations:** Tumour Targeting Group, Division of Genomic Medicine, University of Sheffield Medical School, Beech Hill Road, Sheffield S10 2RX, UK; Cancer Research UK, Molecular Oncology Laboratory, Institute of Molecular Medicine, University of Oxford, Headington, Oxford OX3 9DU, UK

**Keywords:** anoxia, hypoxia, VL30, retrotransposon, HRE, gene therapy

## Abstract

Novel approaches to cancer gene therapy currently exploit tumour hypoxia to achieve transcriptional targeting using oxygen-regulated enhancer elements called hypoxia response elements. The activity of such elements in hypoxic cells is directly dependent on upregulation of the hypoxia-inducible transcription factor-1 However tumours also contain areas of anoxia, which may be considered a more tumour-selective transcriptional stimulus than hypoxia for targeting gene therapy to tumours. Another element, from the rat virus-like retrotransposon, VL30 (termed the ‘secondary anoxia response element’) has been reported to be more highly inducible in rat fibroblasts under anoxia than hypoxia. To investigate anoxia as a potential transcriptional target in human tumours, we have examined secondary anoxia response element inducibility in two human breast cancer cell lines, MCF-7 and T47D, under anoxia, hypoxia and normoxia. In both cell types, the trimerised secondary anoxia response element showed greater inducibility in anoxia than hypoxia (1% and 0.5% O_2_). The anoxic response of the secondary anoxia response element was shown to be dependent on hypoxia-inducible transcription factor-1 and the presence of a hypoxia-inducible transcription binding site consensus (5′-ACGTG-3′). Mutational analysis demonstrated that the base immediately 5′ to this modulates the anoxic/hypoxic induction of the secondary anoxia response element, such that TACGTG>GACGTG>>CACGTG. A similar correlation was found for erythropoietin, phosphoglycerate kinase 1, and aldolase hypoxia response elements, which contain these respective 5′ flanking bases.

*British Journal of Cancer* (2002) **87**, 1173–1181. doi:10.1038/sj.bjc.6600576 www.bjcancer.com

© 2002 Cancer Research UK

## 

Multiple areas of hypoxia and anoxia (i.e. pO_2_ 0–23 mmHg or 0–3% O_2_) exist in malignant tumours (Vaupel *et al*, 1989). Hypoxia induces various transcription factors, including hypoxia-inducible factors (HIFs) 1 and 2 (Wang and Semenza, 1995; Tian *et al*, 1997). These heterodimers consist of α and β subunits, the former being rapidly degraded by the ubiquitin-proteasome system in normoxia (Salceda and Caro, 1997) but not hypoxia. HIF-1 transactivates gene expression in hypoxia by binding to cognate DNA binding sites (consensus sequence 5′-RCGTG-3′) in hypoxia response elements (HREs) present in promoters of corresponding genes (Semenza *et al*, 1996). The HIF-1/HRE system is currently being exploited to achieve hypoxic transcriptional targeting in anti-cancer gene therapies (Dachs *et al*, 1997; Ido *et al*, 2001). However tumours also contain anoxic areas (Vaupel *et al*, 1989), and as some normal tissues also contain mildly hypoxic areas, tumour anoxia could be used as a more specific tumour targeting parameter for gene therapy than hypoxia.

In order to investigate anoxia as a potential target in gene therapy we have examined the activity of an element derived from the rat VL30 element, in two human cancer cell lines. VL30 elements are a multigene family and members of the class of retroviruses and retrovirus-like transposable elements (retroposons or retrotransposons) present in normal rat and mouse DNA (Anderson and Stoler, 1993). Examination of normal rat tissues has demonstrated very low levels of VL30 expression, whereas high levels have been found in rat malignancies, and rat fibroblasts exposed to anoxia (Anderson *et al*, 1989). Induction of the rat VL30 element RNA is markedly different in anoxia *vs* hypoxia, with the former stimulating induction up to 500-fold and hypoxic conditions (0.1–2% O_2_) only a giving a 10-fold induction (Anderson *et al*, 1989).

A 14 base-pair sequence has been shown to mediate the anoxic response of the rat VL30. This sequence demonstrated a greater response in anoxia than in hypoxia (1% O_2_) in primary rat fibroblasts and was therefore termed a ‘secondary anoxia responsive element’ (SARE) (Estes *et al*, 1995). Furthermore, a factor termed the ‘anoxia inducible factor’ (AIF) was shown to be specifically induced in rat fibroblasts under anoxia (Estes *et al*, 1995). This factor was shown to bind to the SARE more predominantly than HIF-1 in anoxia and thus it was suggested that the anoxic response of the SARE was mediated by AIF. However, the anoxic inducibility of the SARE in human cells has not been investigated to date.

Here, we have examined the activity of the SARE in anoxic, hypoxic and normoxic conditions in two human breast cancer cell lines, and compared its inducibility to that of three well-characterised HREs from promoters of the human erythropoietin (EPO), human aldolase (ALD) and murine phosphoglycerate kinase 1 (PGK-1) genes. Since the SARE also contains a HIF-1-binding site (HBS), we used point-mutational analysis, electrophoretic mobility super-shift assays (EMSAs) and a HIF-1α deficient CHO cell line to investigate the importance of HIF-1, the HIF-1 binding site consensus (HBS), and the base immediately 5′ to this, in the anoxic and hypoxic inducibility of the SARE in human cancer cells.

## MATERIALS AND METHODS

### Cell line and culture conditions

MCF-7 cells were obtained from the European collection of cell cultures (ECACC) and maintained in DMEM supplemented with 10% (v v^−1^) foetal calf serum, penicillin (100 U ml^−1^), streptomycin (100 μg ml^−1^), Fungizone (1.25 μg ml^−1^), 4 mM L-glutamine (Gibco), and insulin (2 U ml^−1^) (Human Actrapid, Novo Nordisk). T47D cells were obtained from ECACC and maintained in the same media used for MCF-7 cells, but without addition of insulin. HIF-1α positive and negative Chinese hamster ovary cells were provided by Professor Peter Ratcliffe, Oxford, and maintained as described previously (Wood *et al*, 1998).

### Plasmid construction

Previous reports have demonstrated that multimerising HREs amplifies their response to hypoxia (Firth *et al*, 1994). Thus, for analytical purposes, we have investigated the ability of a SARE trimer to regulate reporter (luciferase; LUC) gene expression in the human breast cancer cell lines, MCF-7 and T47D, in normoxia (21% O_2_), ‘physiological’ hypoxia (4% O_2_), pathological levels of hypoxia (0.5–1% O_2_) and anoxia. We noted that the antisense strand of the SARE contains a HBS (see Table 1a

**Table 1a:**
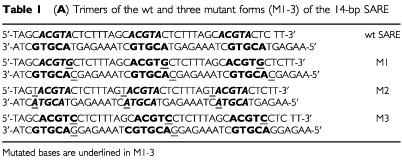
Trimers of the wt and three mutant forms (M1-3) of the 14-bp SARE

), and therefore investigated the relative contributions of this and the putative ‘anoxia-responsive’ 5′-ACGTA-3′ site (Estes *et al*, 1995) to the anoxic and hypoxic inducibility of the SARE, by making three single-base pair SARE mutants in trimerised form (mutants M1-3: see Table 1a). M1 was mutated to replace the putative anoxia-responsive sequence 5′-CACGTA-3′ with a HBS 5′-CACGTg-3′ in the upper strand, and also resulted in a change in the antisense HBS from 5′-TACGTG-3′ to 5′-CACGTG-3′. In M2, the antisense HBS was mutated to 3′–TACGTa-5′, so that the HBS, 5′-ACGTG-3′, was lost and the putative anoxia response sequence, 5′-ACGTA-3′, was present on both strands. In M3, the putative anoxia-responsive sequence, 5′-CACGTA-3′, on the sense strand was changed to 5′-CACGTc-3′, resulting in a change in the antisense strand HBS from 5′-TACGTG-3′ to 5′-GACGTG-3′.

Similarly, trimers of the HREs from the EPO, ALD and PGK-1 genes were also synthesised (monomeric sequences for these are given in Table 1b

**Table 1b:**
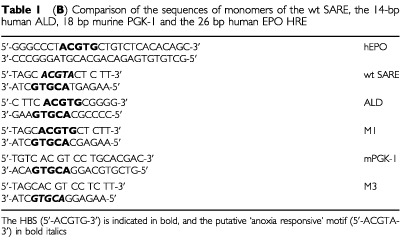
Comparison of the sequences of monomers of the wt SARE, the 14-bp human ALD, 18 bp murine PGK-1 and the 26 bp human EPO HRE

). All trimer oligonucleotides contained a unique *Eco*RI restriction site (at the 5′ end of the sense strand for screening purposes) and *Sac*I and *Kpn*I sites, allowing ligation into the double-restricted pGL3 Promoter vector (Promega), which contains the SV40 promoter (without the SV40 enhancer) upstream of the firefly LUC gene.

### Transient transfections and induction experiments

The Promega Dual LUC system was used to assess reporter gene expression. In this system cells are co-transfected with a pRL plasmid (Renilla LUC driven by a full SV40 promoter), which is used as an internal control. Normalizing the activity of the experimental reporter to the activity of the internal control controls for experimental variability and has been used to represent data concerning HRE activity (Boast *et al*, 1999). Fold inductions were derived from normalized firefly luciferase light units, by dividing the firefly/Renilla luciferase ratio in the test condition (anoxia or hypoxia) by the normoxic firefly/Renilla luciferase ratio (as described by Boast *et al*, 1999). Cells were transfected with a 1 : 40 ratio of Renilla-LUC (control) and firefly LUC-expressing (i.e. test) plasmids, using Fugene-6 (Boehringer Mannheim) according to the manufacturer's protocol. Transfected cells were plated into triplicate wells for each condition in a 24-well plate (30–40×10^3^ cells per well), and then exposed for 16 h to either normoxia (21% O_2_), or hypoxia (0.5, 1 or 4% O_2_, using multigas incubators set at either 4% O_2_/5% CO_2_/91% N_2_, or 1% O_2_/5% CO_2_ /94% N_2_, or 0.5% O_2_/5% CO_2_/94.5% N_2_) or anoxia (5% H_2_/5% CO_2_/90% N_2_ using an anoxic glove-box incubator fitted with a palladium catalyst to scavenge trace O_2_). Oxygen tensions in incubators were independently confirmed using Analox oxygen meters. Cells were then lysed and expression of both types of LUC assayed using the Promega dual luciferase assay kit, and a Dynex MLX microtiter plate luminometer.

### Immunoblot assays

Nuclear extracts were made as described previously (Schreiber *et al*, 1989). Protein levels in these were determined using the Bio-Rad protein assay kit. Antibodies used were: a mouse monoclonal antibody to HIF-1α (Signal Transduction Laboratories), a rabbit polyclonal antibody to HIF-1β (Novus Biologicals), a sheep polyclonal antibody ATF-1 (from Biogenesis Ltd), and a monoclonal antibody to human β-actin (Sigma; used to re-probe stripped blots to control for loading differences in each assay). A mouse monoclonal antibody to human EPAS 1 (HIF-2α) was made in-house (Wiesener *et al*, 1998). Antibodies were used at a final dilution of 1 : 2000, except the EPAS 1 and β-actin antibodies which were used at 1 : 1500 and 1 : 5000. 8% SDS/polyacrylamide gels were used. Proteins were electroblotted onto Hybond-c Super nylon membranes at 40 V for 3 h, and blocked in 5% skimmed milk/0.5% Tween 20 in PBS overnight at 4°C. Incubation with the primary antibody was for 3 h at room temperature (RT). Incubation with the secondary HRP-conjugated antibody in 5% skimmed milk powder in TBS (with 0.05% Tween 20) was then performed for 1 h. Membranes were washed twice in 100 ml TBS/0.05% Tween 20 and twice in 100 ml of TBS, and then incubated in ECL immunoblotting detection reagents (Amersham Pharmacia Biotech) and exposed to X-ray film.

### Electrophoretic mobility shift and supershift assays (EMSAs)

Oligonucleotides for EMSA were synthesised in-house and then treated (each at a concentration of 100 μM) in 10 mM Tris pH 8.5, 50 mM NaCl at 95°C for 5 min and allowed to anneal by cooling to room temperature. Radiolabelling was carried out by end-filling of the overhanging thymidines at each 5′ end using Klenow polymerase (Life Technologies, Paisley, UK) to add ^32^P labelled dATP ([α^32^P]dATP). Labelling reaction was carried out in a total volume of 50 μl containing approximately 0.12 μM probe, 1× reaction buffer (Life Technologies, Paisley, UK), 1 μl of 10 mM dNTPs (excluding dATP), 2 μl [α^32^P]dATP (370 MBq ml^−1^) 1 μl Klenow polymerase (1 U μl^−1^). The labelling reaction mix was incubated for 15 min at 30°C. The labelled probe was then washed, removing unincorporated [α^32^P]dATP by using the QIAquick Nucleotide Removal Kit (Qiagen, Sussex, UK) according to the manufacturer's protocol with a minor change which included an additional wash of the labelled probe. The labelled probe was then eluted in 50 μl of EB (Tris-Cl, pH 8.5) and the purity was assessed by running 1 μl of the probe on a thin layer chromatography (TLC) plate (Merck, Lutterworth, UK) using a TLC separation buffer (1.2 M HCl and 0.8 M ammonium acetate), and the purity of the labelled probe visualised by autoradiography.

In each binding reaction, 3–4 μg of nuclear extract was used. A ‘null SARE’ oligonucleotide (a mutated form of the SARE lacking a 5′-CGTG-3′ HIF-1 binding site consensus) was used as a competitor at an excess of 100–200 fold (μM) over the radiolabelled SARE or mutant SARE probes. The probe and competitor were pooled and 0.87 μl used for each binding/competition assay (which was carried out for 15 min at room temperature). Binding was carried out in 10 mM Tris pH 8.0, 50 mM NaCl, 5 mM EDTA, 4% glycerol, with 2.5 mM DTT (added just before use) in a total volume of 20 μl.

Sequences of the oligonucleotides used for the production of probes and competitors were as follows (overhanging T's and unique *Eco*RI site shown in bold; monomers separated by a bar; mutated bases underlined):

ARE trimer probe:

5′-**TTT**TAGCACGTACTCTT TAGCACGTACTCTT TAGCACGTACTCTT**GAATT**C-3′.

SARE null competitor trimer:

5′-**TTT**TAGCTATTACTCTT TAGCTATTACTCTT TAGCTATTACTCTT**GAATTC**-3′.

For supershift assays, 1–2 μg antibody (to HIF-1α, HIF-1β, EPAS 1, or ATF-1) was added to the binding reaction after 15 min and incubated at room temperature for a further 30 min before separation on a 3% non-denaturing polyacrylamide gel run in 0.5× TBE buffer/300 v at 4°C. The gel was then run for 1.5 h. Extracts were made as explained for immunoblots.

### Statistics

**Figure 1:**
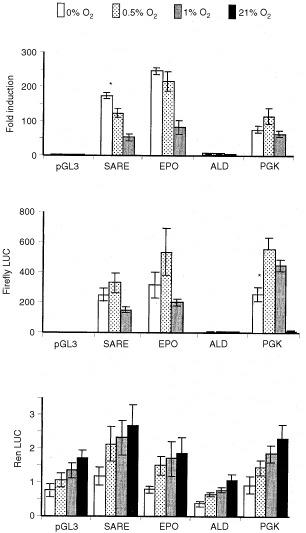
Mean (±s.e.m.) fold induction (relative to normoxia; top panel), firefly LUC (middle panel) and Renilla LUC (bottom panel) detected in MCF-7 cells following transfection with the pGL3 Promoter vector, or this containing trimerised versions of the SARE, the human EPO HRE, the human ALD HRE or the murine PGK-1 HRE. Cells were exposed to 0, 0.5, 1 or 21% O_2_ for 16 h. *P<0.05 with respect to the same trimer at 0.5% O_2_ (unpaired *t-*test). Pooled data from three experiments are shown.

An unpaired *t-*test was used to compare fold induction and/or LUC values obtained for each trimer (i.e. at different O_2_ levels) in Figures 1 –

4

**Figure 4:**
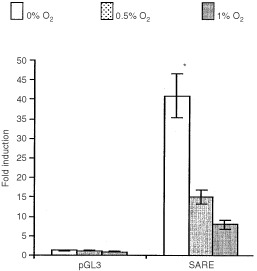
Mean (±s.e.m.) fold induction in T47D cells following transfection with either the pGL3 Promoter vector alone or this containing a trimer of the wtSARE following exposure to 0, 0.5, or 1% O_2_ for 16 h. *P<0.05 with respect to the same trimer at 0.5% O_2_ (unpaired *t*-test). Pooled data from four experiments are given.

.

## RESULTS

### Comparison of the SARE with various HREs at different oxygen tensions

No induction was seen for the pGL3 Promoter plasmid in hypoxia/anoxia, but the SARE-LUC construct was highly inducible in both hypoxia and anoxia. Normalised LUC induction values relative to normoxia (21% O_2_) were approximately 170-fold in anoxia, 125-fold in 0.5% O_2_, and 60-fold in 1% O_2_. The EPO HRE trimer gave even higher fold inductions: 250-fold (anoxia), 215-fold (0.5% O_2_) and 80-fold (1% O_2_). The patterns of induction of the SARE and EPO were similar, being highest in anoxia and decreasing slightly in 0.5%, but more markedly in 1% O_2_ (Figure 1). There was a significant (*P*=0.003) difference between the fold induction of the SARE (but not EPO HRE) in anoxia and 0.5% O_2_. Both trimers however, showed a significant (*P*<0.05) drop in 1% O_2_ relative to anoxia. Due to its higher normoxic activity, the PGK-1 HRE showed lower fold inductions than the SARE or EPO trimers, particularly in anoxia, and showed no significant difference in fold induction values between anoxia, 0.5% and 1% O_2_. The ALD HRE trimer construct was the least inducible, giving similar values in anoxia to those in 0.5 and 1% O_2_ (3–5 fold induction).

Although HRE activity has generally been represented as normalised reporter gene expression (Boast *et al*, 1999), one has to be cautious about resultant artefacts if the internal control expression decreases in a particular experimental condition. For example, the level of Renilla LUC produced under different O_2_ tensions contributes to the magnitude of the firefly luciferase/Renilla LUC ratios and hence the fold induction data. Renilla LUC values showed a marked progressive decrease as the oxygen levels decreased, dropping by as much as 30–40% under anoxia (Figure 1), an observation also reported by Dachs *et al* (2000). This may reflect transcriptional/translational shutdown during oxygen deprivation (Tinton *et al*, 1997). Although fold induction values for the SARE and EPO were highest in anoxia, levels of firefly LUC expression peaked at 0.5% O_2_. As Renilla LUC levels were lower in anoxia, this gives the appearance of greater expression from the SARE and EPO constructs in anoxia than hypoxia. Although the levels of expression from the SARE- and EPO-driven firefly LUC constructs were slightly reduced in anoxia relative to 0.5% O_2_, the decrease was not statistically significant. PGK-driven firefly LUC expression dropped significantly (*P*=0.02) between 0.5% O_2_ and anoxia (Figure 1).

However, the trend in which the internal control responded to the various O_2_ levels was similar between various experiments. In all experiments, the Renilla luciferase light units dropped approximately 2.5-fold in anoxia compared to normoxia. Furthermore, the Renilla luciferase light units in anoxia were not significantly (*P*=0.2) different between different test groups, so the fold induction data suggest that the SARE was, indeed, further activated in anoxia than in 0.5% O_2_.

### Activity of the SARE, EPO and PGK-1 HREs in mild (i.e. physiological) hypoxia

**Figure 2:**
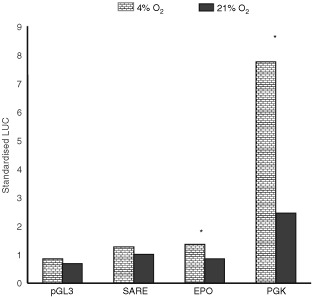
Mean (±s.e.m) standardised LUC light units (ratio of firefly LUC/Renilla LUC readings) detected in MCF-7 cells following transfection with the pGL3 promoter vector or this containing trimerised versions of the SARE, the human EPO HRE, the human ALD HRE, the murine PGK-1 HRE. Cells were exposed to 4 or 21% O_2_ for 16 h. *P<0.01 with respect to same trimer at 21% O_2_ (unpaired *t*-test). Pooled data from three experiments are shown.

The above findings and previous reports of the rat VL30 being silent in 5% O_2_ (Anderson *et al*, 1989) suggest that the SARE and EPO HRE constructs may be more selective for low levels of oxygen than the PGK-1 construct. We therefore examined the responses of the SARE, EPO HRE and PGK-1-driven reporter constructs to mild hypoxia (4% O_2_) (Figure 2

). The PGK-1 HRE, but not the SARE or EPO HRE, was significantly (*P*<0.0001) induced in this oxygen tension.

### Use of mutagenesis to investigate the function of the SARE under anoxia and hypoxia

**Figure 3:**
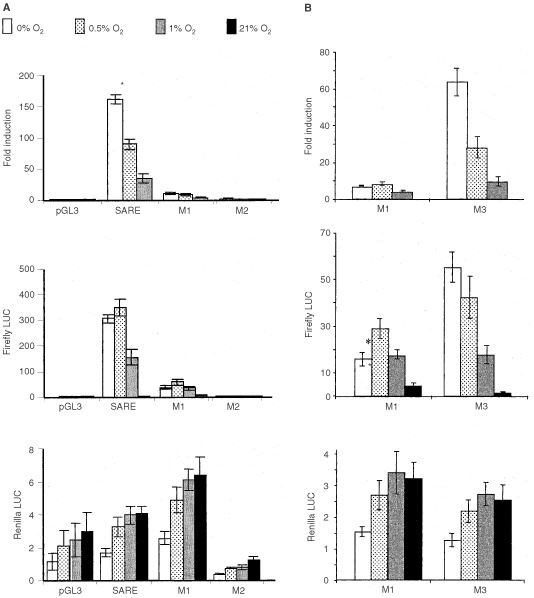
(**a**) Mean (±s.e.m.) fold induction (top panel), firefly LUC (middle panel) and Renilla LUC (bottom panel) detected in MCF-7 cells following transfection with either the pGL3 Promoter vector or this containing trimerised versions of the wt SARE or mutant forms of this (M1 and M2; see Table 1a,b). Cells were exposed to 0, 0.5, 1 or 21% O_2_ for 16 h. *P<0.05 with respect to the same trimer at 0.5% O_2_ (unpaired *t*-test). Pooled data from four experiments are given. (**b**) Mean (±s.e.m.) fold induction (top panel), firefly LUC (middle panel) and Renilla LUC (bottom panel) in MCF-7 cells following transfection with either the pGL3 Promoter vector alone or this containing trimers of M1 or M3 following exposure to 0, 0.5, 1, or 21% O_2_ for 16 h. *P<0.05 with respect to the same trimer at 0.5% O_2_ (unpaired *t*-test). Pooled data from four experiments are given.

When trimerised, the SARE was induced 40-, 90- and 160-fold (relative to normoxia) in MCF-7 cells (Figure 3i

), and eight-, 15- and 40-fold in T47D cells (Figure 4) in 1%, 0.5% and anoxia respectively. No induction was seen for the pGL3 Promoter plasmid in hypoxia or anoxia. In anoxia, the SARE trimer produced significantly (*P*=0.008) higher fold induction than in 0.5% O_2_ (Table 2

**Table 2:**
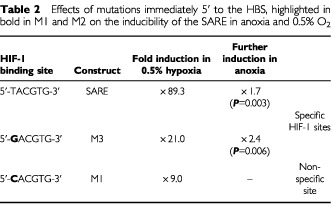
Effects of mutations immediately 5′ to the HBS, highlighted in bold in M1 and M2 on the inducibility of the SARE in anoxia and 0.5% O_2_

). In contrast to these fold inductions, which were derived from standardised mean relative light units (Firefly LUC values standardised by expression of Renilla LUC), there was no significant difference between the SARE-driven firefly LUC values in 0.5% and anoxia.

M1 resulted in markedly (>10-fold) lower levels of induction than the SARE (Figures 3a,b). M2 showed virtually no induction in hypoxia or anoxia (Figure 3a). M3 responded with a similar trend to the SARE in hypoxia and anoxia, albeit with a lower induction (>four-fold induction difference), but significantly (*P*=0.006) higher induction than M1 in anoxia and hypoxia (Figure 3a,b) (Table 2).

### Analysis of transcription factor expression and binding

**Figure 5:**
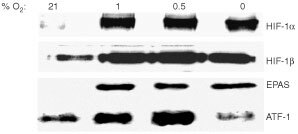
Immunoblot analysis of HIF-1α, HIF-1β, EPAS 1, and ATF-1 in MCF-7 cells following exposure to 0, 0.5, 1, and 21% O_2_ (normoxia) for 16 h. Blots were stripped and re-probed for β-actin as a loading control; no differences were observed (data not shown).

**Figure 6:**
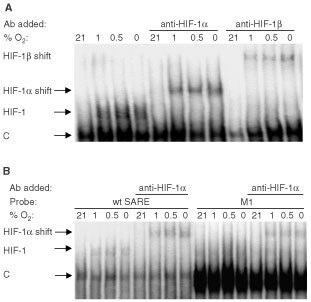
(**a**) Electrophoretic mobility supershift assay showing binding of HIF-1α and HIF-1β in MCF-7 extracts to the trimerised SARE probe following exposure of cells for 16 hours to various O_2_ tensions. Bands appear in 1, 0.5 and 0% O_2_. These inducible complexes were supershifted in the presence of a monoclonal antibody to HIF-1α or a polyclonal antibody to HIF-1β (C=binding of constitutive factors). (**b**) Electrophoretic mobility supershift assay showing the inducible HIF-1 band binding to the wt SARE or M1 trimers (the HIF-binding sequence of the latter being 5′-CACGTG-3′) in nuclear extracts of MCF-7 cells exposed for 16 h to various O_2_ tensions. Bands appear in 1, 0.5 and 0% O_2_ with both probes. These are super-shifted by addition of a monoclonal antibody to HIF-1α. More extensive binding of constitutive factors (‘C’) can be seen with M1 than with the wt SARE probe.

Low levels of HIF-1α and HIF-1β were detectable in normoxic MCF-7 nuclear extracts. No HIF-2α (EPAS 1) was detected in normoxia. Levels of all three of these proteins increased markedly in hypoxia (1% and 0.5% O_2_) and anoxia (Figure 5

). As several members of the bZIP (basic/leucine zipper domain) family of transcription factors such as ATF-1 have been implicated in the regulation of HRE activity, and shown to bind to the HBS in certain HREs (Kvietikova *et al*, 1995), we also examined via immunoblot analysis the level of ATF-1 protein in normoxia, hypoxia and anoxia. In addition, via electrophoretic mobility supershift assays, we also examined whether ATF-1 binds the SARE. ATF-1 was present in normoxia, elevated at 1% and 0.5% O_2_, but reduced in anoxia in MCF-7 cells (Figure 5). Figure 6a

shows that HIF-1 bound to the radiolabelled SARE trimer at 0.5, 1 and 0% O_2_ in EMSAs, and was supershifted by monoclonal antibodies to HIF-1α and HIF-1β.

The HIF-1α monoclonal antibody also supershifted M1 (Figure 6b) and M3 probes (not shown). This is as expected since SARE, M1 and M3 contain the HBS (5′-ACGTG-3′). Binding of undefined constitutive factors was more extensive with the M1 than the SARE, M 2 or M3 trimers (Figure 6b).

**Figure 7:**
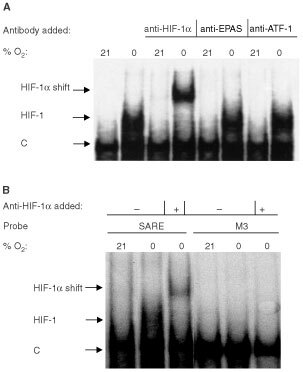
(**a**) Electrophoretic mobility supershift assay showing HIF-1 binding to wt SARE in nuclear extracts of MCF-7 cells exposed for 16 h to anoxia. The inducible HIF-1 band is super-shifted by addition of a monoclonal antibody to HIF-1α. Addition of the antibody to EPAS 1 (HIF-2α or ATF-1 failed to supershift the constitutive factors (‘C’) or the inducible band in anoxia. (**b**) Electrophoretic mobility supershift assay showing HIF-1 binding to wt SARE in nuclear extracts of MCF-7 cells exposed for 16 h to anoxia. The inducible HIF-1 band is super-shifted by addition of a monoclonal antibody to HIF-1α. M2 (which lacked the HBS) failed to bind the inducible HIF-1 in the assay conditions used.

Antibodies to HIF-2α (EPAS 1), and ATF-1 did not supershift the complex bound to the SARE trimer (Figure 7a

). No HIF-1 binding was observed for the M2 probe, which lacks a HBS (Figure 7b).

### Anoxic and hypoxic induction of the SARE in HIF-1*α* knockout cells

**Figure 8:**
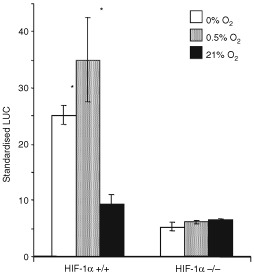
Mean (±s.e.m.) standardised LUC light units (ratio of firefly LUC/Renilla LUC readings) in either wild type (HIF-1 +/+) or HIF-1α knockout (HIF-1 −/−) CHO cells (Wood *et al*, 1998) following transfection with a pGL3 Promoter plasmid expressing firefly LUC under control of a trimer of the SARE, following exposure to 21% O_2_, 0.5% O_2_, or 0% O_2_ for 16 h. The SARE inducibility is lost in the HIF-1α knockout (HIF-1 −/−) CHO cells. *P*=0.003 with respect to HIF-1 +/+ cells at 21% O_2_, or the same oxygen tensions for HIF−/− cells.

In wild type CHO cells, the SARE was induced in both anoxia and 0.5% O_2_. However, no SARE induction was observed in anoxia or 0.5% O_2_ in HIF knockout CHO cells (Figure 8

).

## DISCUSSION

Here we show for the first time that the SARE is a potent HRE with inductions (as an average of two sets of experiments, presented as Figures 1 and 3) of 165-, 107-, and 50-fold in anoxia, 0.5, and 1% oxygen respectively in the human breast cancer cell line MCF-7 *in vitro*. A similar pattern of SARE induction was seen in another human breast cancer cell line, T47D. Thus, in contrast to the other HRE constructs the SARE responded approximately 1.5-fold more in anoxia than in 0.5% O_2_ in MCF-7 cells. In order to evaluate the potential role of the HBS (5′-ACGTG-3′), and the ‘HIF-1-like anoxia-responsive’ sequence 5′-ACGTA-3′ (Estes *et al*, 1995), in the anoxic inducibility of the SARE, we created single point mutations of the SARE: M1, M2, and M3. In M1, the putative anoxia-responsive sequence (5′-ACGTA-3′) was ablated, producing two identical HBS (with the sequence 5′-CACGTG-3′), one on each strand, and changing the HIF site on the antisense strand from the highly inducible 5′-TACGTG-3′ to the less inducible 5′-CACGTG-3′ (Kimura *et al*, 2001). In accordance with their findings, M1 responded poorly to hypoxia and anoxia.

In M2, the HBS on the antisense strand was ablated, creating two putative ‘anoxia-responsive’ 5′-ACGTA-3′ sequences on opposing strands. This did not bind HIF-1 in our EMSAs or show inducibility in hypoxia or anoxia, indicating that the sequence 5′-ACGTA-3′ is not sufficient for hypoxic or anoxic induction of the SARE. The Renilla luciferase (internal control) for M2 demonstrated lower values than the Renilla luciferase plasmid for the other constructs (Figure 3). This was probably due to the fact that the experiments with M2 were performed on separate days – further confirmed by the fact that the Renilla luciferase values for SARE and pGL3 transfections performed on the same day as the M2 group also demonstrated lower values than the ones seen in Figure 3. Despite these lower Renilla values, the SARE demonstrated the usual pattern of induction as observed in Figures 3 and 4 (data not shown). Thus, the low Renilla values of M2 did not account for its lack of inducibility.

To investigate the functionality of a SARE lacking the putative ‘anoxia-responsive’ 5′-ACGTA-3′ sequence, but with a more highly inducible HBS than the 5′-CACGTG-3′ sequence present in M1, M3 was constructed. In this, the 5′-ACGTA-3′ was mutated to 5′-ACGTc-3′, resulting in a change in the antisense strand HIF site from the original 5′-TACGTG-3′ to 5′-GACGTG-3′. In agreement with the findings of Kimura *et al* (2001), M3 demonstrated activity intermediate between the SARE (5′-TACGTG-3′) and M1 (5′-CACGTG-3′). Although firefly LUC expression in anoxia and severe hypoxia (0.5% O_2_) was lower relative to normoxia for M3 than for the wild type SARE, this mutant was more selectively inducible in anoxia and severe hypoxia (0.5% O_2_), relative to 1% O_2_, than the SARE. The bases immediately 5′ to the HBS in the SARE, M1 and M3 are identical to those in the EPO, ALD, and PGK-1 HREs respectively. Trimers of these HREs demonstrated a similar trend of induction to the SARE, M3 and M1 (i.e. EPO>PGK>ALD; TACGTG>GACGTG>CACGTG; SARE> M3>M1). This accords well with functional studies of such sequences in the promoters of hypoxia-responsive genes (Semenza *et al*, 1996; Ebert *et al*, 1998), and also confirms the recent findings of Kimura *et al* (2001) for the VEGF HRE, and thus may be a common characteristic of HREs. In particular, the poor hypoxic and anoxic inducibility of M1 compared to the SARE (from which it differs only in the base immediately 5′ to the HBS), shows the importance of this base in determining the inducibility of the SARE at low O_2_ levels. The HBS in M1 is the same as the HBS (5′-CACGTG-3′) present in the ALD gene. Interestingly, the ALD HBS (5′-CACGTG-3′), which was poorly responsive in the context of the SARE, has been shown previously to be non-responsive to hypoxia (1% O_2_) in the context of the native ALD promoter (Semenza *et al*, 1996). Thus, the data from the point mutation analysis demonstrate that the function of the SARE in hypoxia and anoxia is dependent on a highly inducible antisense HBS. Interestingly, this HBS (including flanking base sequences, i.e. 5′-TACGTGCT-3′) is identical to the one present in the hEPO HRE sense strand, and both elements responded similarly to hypoxia and anoxia.

A previous report by Ebert *et al* (1998) mentioned the importance of the 5′ flanking base of an HRE on the affinity of the HBS for HIF-1. However our EMSA and induction data suggest that it is not the binding affinity of these sites for HIF-1 that is affected, but rather the ability of bound HIF-1 to activate transcription at these different sites. M1, despite having two core HBS (5′-CGTG-3′), and HIF-1 binding in EMSAs similar to that of the SARE, is barely inducible, giving approximately 10-fold lower levels of hypoxia-inducible transcriptional activation. This suggests that either the mutation of the 5′-CGTA-3′ sequence to 5′-CGTg-3′, or the concomitant change from a 5′-TACGTG-3′ to a 5′- cACGTG-3′ HBS on the antisense strand, while not affecting the overall amount of HIF-1 binding, affects the ability of this factor to activate transcription. The base 5′ to the HBS (5′-ACGTG-3′) could be involved in the binding of an accessory protein, alter the conformation and activity of HIF-1 bound to the site, or selectively bind different post-translationally modified forms of HIF-1. Furthermore, our EMSA results suggest that these flanking bases may influence the specificity of the binding site for HIF-1. The poorly inducible M1 (5′-cACGTG-3′) site showed greater binding to constitutive proteins than the SARE or M3, which could explain why this mutant was so poorly inducible by hypoxia and anoxia compared to the SARE and M3. Indeed, 5′-CACGTG-3′ is a binding site for a number of other transcription factors such as USF, c-Myc/Max and Rox/Max heterodimers, or a homodimer of HIF-β (ARNT) (Meroni *et al*, 1997; Swanson and Yang, 1999), some of which repress transcription upon binding. Similar levels of HIF-1α were seen at 1% O_2_, 0.5% O_2_, and anoxia, and the EMSA indicated no major difference in HIF-1 binding to the SARE trimer between these oxygen levels. EPAS 1 and ATF-1 were also present in extracts of hypoxic and anoxic MCF-7 cells, but our EPAS 1 and ATF-1 antibodies failed to super-shift the protein complexes bound to the wt SARE probe.

The feasibility of using HRE-targeted gene therapy to treat tumours systemically is likely to depend on: (1) high-level gene expression in the severely hypoxic/anoxic sites in tumours; and (2) no or low expression in normoxia and/or physiological hypoxia. We show that the SARE is a variant form of HRE that is highly inducible in severe hypoxia and anoxia, with negligible activity in mild physiological hypoxia (i.e. 4% O_2_) in human MCF-7 cells.

The differences between the inducibility of the SARE and its mutant, M3, may be important for gene therapy. M3 demonstrated higher fold induction in severe hypoxia/anoxia, relative to mild hypoxia (1% O_2_) than the SARE. This measure of hypoxia specificity is more relevant in terms of hypoxia-targeted gene therapy than fold induction relative to 21% oxygen, which is super-physiological. Interestingly, the absolute levels of LUC expression from the SARE showed little difference between 0–1% O_2_, suggesting that the level of expression is maintained at levels of oxygen below 1%, rather than being induced further by lower oxygen levels. It is likely that M3 would be more suitable than the SARE for highly targeted gene expression in severely hypoxic and anoxic sites, as it would produce lower levels of expression in non-diseased, mildly hypoxic tissues (i.e. containing 1% O_2_). However, M3 has a drawback; despite its greater specificity for severe hypoxia/anoxia, the absolute levels of LUC expression were approximately six-fold lower than those seen with the SARE in these conditions. This could perhaps be compensated for by increasing transfection efficiency or copy number, by combining with a stronger promoter such as CMV, or by the production of constructs containing multiple copies of a therapeutic gene driven by M3. The choice of the HRE for systemic gene delivery would eventually also depend on the type of vector used. An efficient tumour-targeting vector (Ameri and Wagner, 2000; Kircheis *et al*, 2001) would widen the choice of HREs by providing an extra level of targeting which could by-pass certain tissues/organs that may contain low levels of oxygen (Kircheis *et al*, 2001). Further work is now warranted to demonstrate which of the HREs described here have utility in such novel forms of gene therapy *in vivo*.
